# The Utility of Automated ASPECTS in Acute Ischemic Stroke for Intravenous Recombinant Tissue Plasminogen Activator (IV-rtPA) Therapy

**DOI:** 10.3390/neurolint14040077

**Published:** 2022-11-21

**Authors:** Soichiro Shibata, Kenzo Sakurai, Keiji Tachikawa, Riyoko Ko, Sakae Hino, Takayuki Fukano, Kenji Isahaya, Takafumi Haraguchi, Junji Yamauchi, Kenichiro Tanabe, Misako Nagasaka, Yuta Hagiwara, Takahiro Shimizu, Hisanao Akiyama, Yasuyuki Kobayashi, Yasuhiro Hasegawa, Yoshihisa Yamano

**Affiliations:** 1 Division of Neurology, Department of Internal Medicine, St. Marianna University School of Medicine, Kawasaki 216-8511, Japan; 2 Department of Advanced Biomedical Imaging and Informatics, St. Marianna University School of Medicine, Kawasaki 216-8511, Japan; 3 Department of Rare Diseases Research, Institute of Medical Science, St. Marianna University School of Medicine, Kawasaki 216-8511, Japan; 4 Department of Frontier Medicine, Institute of Medical Science, St. Marianna University School of Medicine, Kawasaki 216-8511, Japan; 5 Division of Hematology and Oncology, Department of Medicine, University of California, Irvine School of Medicine, Orange, CA 92697, USA; 6 Department of Medical Information and Communication Technology Research, St. Marianna University School of Medicine, Kawasaki 216-8511, Japan; 7 Division of Neurology, Department of Internal Medicine, Shin-Yurigaoka General Hospital, Kawasaki 215-0026, Japan

**Keywords:** artificial intelligence, recombinant tissue plasminogen activator therapy, ASPECTS, acute cerebral infarction

## Abstract

Purpose: This study aimed to investigate the accuracy and clinical significance of an artificial intelligence (AI)-based automated Alberta Stroke Program Early Computed Tomography (ASPECT) scoring software of head CT for the indication of intravenous recombinant tissue plasminogen activator (rt-PA) therapy. Methods: This study included two populations of acute ischemic stroke: one comprised patients who had undergone head CT within 48 h of presentation (Population #1, *n* = 448), while the other included patients within 4.5 h from onset (Population #2, *n* = 132). The primary endpoint was the concordance rate of ASPECTS of the neurologists and AI software against the benchmark score. The secondary endpoints were to validate the accuracy of the neurologist and AI software in assessing the ability to rule out extensive infarction (ASPECTS of 0–5) in population #2. Results: The reading accuracy of AI software was comparable to that of the board-certified vascular neurologists. The detection rate of cardiogenic cerebral embolism was better than that of atherothrombotic cerebral infarction. By excluding extensive infarction, AI-software showed a higher specificity and equivalent sensitivity compared to those of experts. Conclusions: The AI software for ASPECTS showed convincing agreement with expert evaluation and would be supportive in determining the indications of intravenous rt-PA therapy.

## 1. Introduction

Stroke is not only the second leading cause of mortality but also the third leading cause of long-term care in the world [1,2]. To improve the outcomes of patients with cerebral infarction, it is important to determine suitability for intravenous recombinant tissue plasminogen activator (rt-PA) therapy and for endovascular therapy in the acute phase, because these therapies have been proved to be effective in patients with acute ischemic stroke [3]. In acute cerebral infarction, rt-PA therapy should be performed within 4.5 h from the onset of symptoms, while endovascular therapy is recommended within 6 h from symptom onset [3]. However, there are significant regional differences in acute phase treatment. For instance, in Japan, the percentage of intravenous rt-PA therapy for cerebral infarction ranges from 0% to >60% among hospitals. In addition, the number of patients treated with endovascular therapy is low, at <3 per 100,000 people per year in four out of 47 prefectures [4,5]. Thus, there are still many hospitals where cerebral infarction is treated with conservative therapy alone [6]. To achieve standardization of treatment in the acute phase of cerebral infarction, diagnosis and treatment should be improved, especially in non-urban areas [7].

Among acute stroke treatments, intravenous rt-PA therapy can theoretically be administered by non-stroke physicians if they can accurately read the head computed tomography (CT) scan images [6]. To guide the indication for intravenous rt-PA therapy, the Alberta Stroke Programme early CT score (ASPECTS) [8], a 10-point quantitative topographic CT scan score, is widely used. ASPECTS is a tool for assessing the extent of early ischemic changes in 10 regions of the middle cerebral artery territory [8]. However, scoring ASPECTS using head CT images of acute cerebral infarction is not easy, and the reading accuracy is low if the physician’s experience is limited [9]. Moreover, there is a possibility that non-specialists may not be comfortable in determining the indication for intravenous rt-PA therapy due to difficulties in scoring ASPECTS by CT image reading. Thus, some patients do not receive appropriate treatment.

As a means of addressing such issues, a head CT image reading software utilizing artificial intelligence (AI) was recently developed, and its reading accuracy of ASPECTS was found to be equivalent to that of stroke specialists in cerebral infarction [10,11,12,13,14]. However, studies have examined the usefulness of the software in determining the indication for endovascular therapy, mainly in cases where the target patient underwent endovascular therapy or in cases of large vessel occlusion (LVO) [14,15,16]. In routine clinical practice, physicians should examine all types of ischemic stroke and identify patients who are candidates for t-PA treatment. Thus, there is still insufficient data on its accuracy based on the type of stroke and the clinical significance in determining the indication for intravenous rt-PA therapy (ASPECTS of ≥6) in cerebral infarction [17]. Therefore, the current study aimed to analyze the accuracy and clinical significance of the automated ASPECT scoring software using AI (hereinafter referred to as AI software) for patients with acute cerebral infarction of various types, including non-LVO patients, and for the indication of intravenous rt-PA therapy in patients with cerebral infarction.

## 2. Materials and Methods

### 2.1. Participants

There were 500 consecutive patients with acute ischemic stroke admitted to our hospital between October 2017 and December 2020. The selection criteria were as follows: (1) patients who had undergone head CT scan and head MRI within 48 h of presentation, (2) those who had undergone neurological examination including National Institutes of Health Stroke Scale (NIHSS) performed by a neurologist at presentation, and (3) those with confirmed data on onset time or the time the patient was last known to be well. The exclusion criteria were as follows: (1) patients whose images were difficult to read because of artifacts and unclear or incomplete images; and (2) those with images in which the analysis processing by the AI software could not be performed adequately (due to some missing image data).

### 2.2. Data Source and Assessment

The following data were collected retrospectively from medical records. The clinical information included age, sex, blood pressure at presentation, level of consciousness, presence of headache and motor palsy including paralyzed side, NIHSS score, time from stroke onset to CT scan, and history of intravenous rt-PA therapy and endovascular therapy. Imaging data were collected via head CT scan and subsequent head MRI.

**Figure 1:**
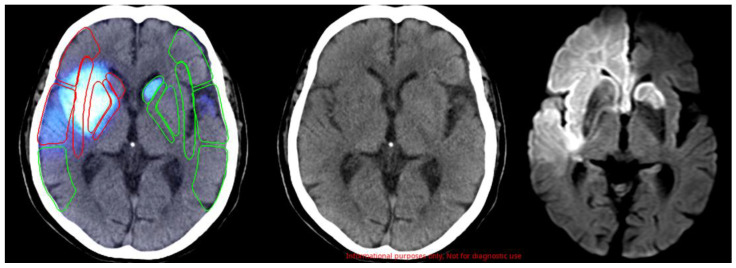
Representative images of ischemic stroke by AI software. From left to right: images assessed using the AI software, non-contrast head CT scan, and diffusion-weighted head MRI. In the AI software image, the red framed areas indicate early ischemic changes and the green framed areas indicate non-ischemic changes.

To determine the ASPECTS benchmark score (BS) criteria, a board-certified vascular neurologist and a radiological specialist performed the ASPECT scoring of head CT scan images with reference to head MRI images. If there was a discrepancy, the scores were determined via a discussion, and these scores were used as the BS criteria. Next, the head CT images were read by six neurologists (board-certified vascular neurologists (physicians A and B)), neurology fellows (physicians C and D), and neurology residents (physicians E and F), who were different from the physicians who created the BS criteria. Six neurologists scored the ASPECTS results using only information on motor paralysis (right hemiplegia or left hemiplegia or unknown). In addition, the head CT images were read using the AI software (Abierto Reading Support Solution for Stroke; Canon Medical Systems Corporation, Tochigi, Japan). A representative image is shown in Figure 1.

### 2.3. Endpoints

This study included two populations: one comprised patients with acute ischemic stroke who had undergone head CT and head MRI within 48 h of presentation irrespective of the time from onset (Population #1). The other included patients with acute ischemic stroke who had undergone head CT within 4.5 h from onset (Population #2). The primary endpoint for each population was the concordance rate of ASPECTS of the neurologists and AI software against the BS criteria in all types and in each type of ischemic stroke. The secondary endpoints were used in population #2 to validate the accuracy of the neurologist and AI software in assessing the ability to rule out extensive infarction, which was defined as an ASPECTS of 0–5.

### 2.4. Statistical Analysis

Patient characteristics were summarized using means and standard deviations (SD) for continuous variables and proportions for categorical variables. To determine if the NIHSS showed normal probability distribution, we conducted the Shapiro-wilk test using R version 4.2.0. (R Foundation, Vienna, Austria). To analyze the correlations of the ASPECTS against the BS criteria between the six physicians (A to F) and AI software, we analyzed the intraclass correlation coefficient (ICC: (2,1)) using SAS version 9.4 (SAS Institute, Cary, NC, USA). As for the criteria for judging the ICC score, we utilized the following criteria: values less than 0.5 were indicative of poor reliability, values between 0.5 and 0.75 indicated moderate reliability, values between 0.75 and 0.9 indicated good reliability, and values greater than 0.90 indicated excellent reliability [18].

## 3. Results

### 3.1. Patient Characteristics

**Figure 2:**
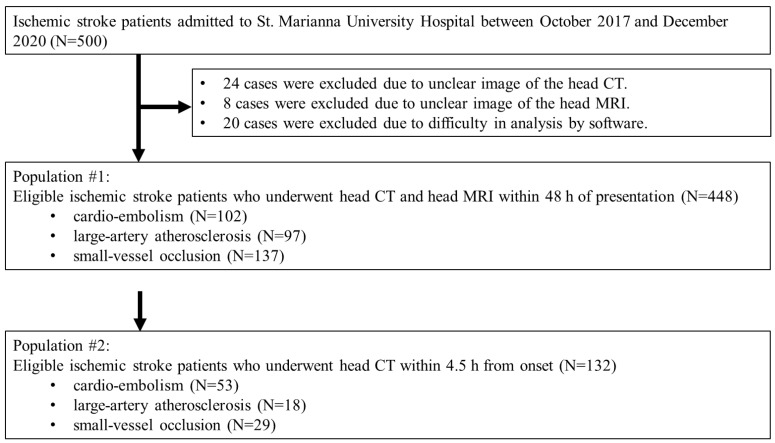
Study flow chart.

Of 500 patients, 24 were excluded due to unclear or incomplete head CT images, 8 due to unclear or incomplete head MRI images, and 20 due to inadequate image acquisition that prevented analysis using the AI software. As a result, 448 patients were included as Population #1 and 132 patients as Population #2 (Figure 2). In populations #1 and #2, 60.8% and 57.6% were men, with a mean age of 73.8 ± 13.2 and 74.8 ± 13.0 years, respectively. In total, 364 (81.3%) and 107 (81.1%) patients presented with unilateral motor palsy, median NIHSS was 3 (interquartile ranges: IQR, 1–8), 300 (67.0%) and 75 (56.8%) with NIHSS ≤ 5, 95 (21.2%) and 30 (22.7%) with 6 ≤ NIHSS ≤ 15, and 53 (11.8%) and 27 (20.5%) with 16 ≤ NIHSS, respectively. In total, 137 (30.6%) and 29 (22.0%) patients presented with lacunar infarction, 96 (21.4%) and 18 (13.6%) with atherothrombotic stroke, 102 (22.8%) and 53 (40.2%) with cardiogenic cerebral embolism, respectively. Furthermore, 20 (4.5%) and 20 (15.2%) patients received intravenous rt-PA therapy and 4 (0.9%) and 4 (3.0%) patients received endovascular therapy, respectively. The mean time from stroke onset to CT scan imaging was 18.9 ± 18.3 h and 2.4 ± 1.0 h, respectively (Table 1).

### 3.2. Primary Outcome

**Figure 3:**
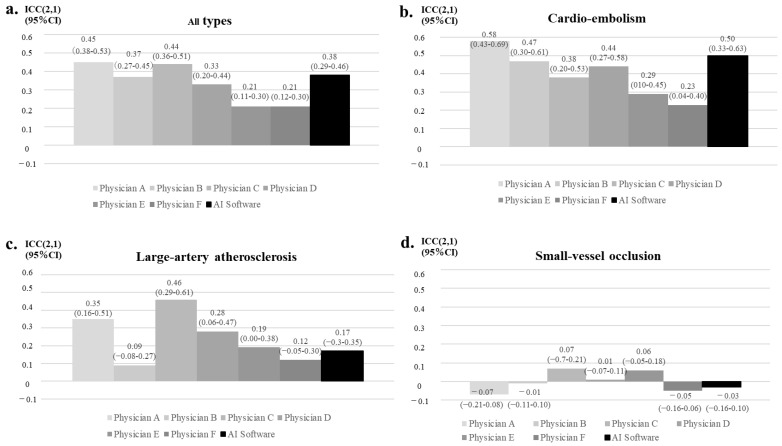
The reading accuracies of the AI software and neurologists in patients who had undergone CT images within 48 h of presentation. The reading accuracies of the AI software and neurologists against the BS criteria for all types of ischemic stroke patients (**a**), for cardio-embolism (**b**), for large-artery atherosclerosis (**c**), and for small-vessel occlusion (**d**). The type of physician is shown on the X axis; board-certified vascular neurologists (physicians A and B), neurology fellows (physicians C and D), and neurology residents (physicians E and F). The inter-assessor reliability of the total ASPECTS is shown in Y axis.

In population #1, the AI software had a better trend compared with the neurology resident (physicians E and F), showing a level comparable to those of the board-certified vascular neurologists (physicians A and B) and neurology fellows (physicians C and D) (Figure 3a). According to the type of ischemic stroke, the highest rate of agreement was observed for cardiogenic cerebral embolism (Figure 3b–d). In cardiogenic cerebral embolism, the results from the AI software were comparable to those of the board-certified vascular neurologist (physicians A and B) and were better than those of the neurology fellows (physicians C and D) and neurology residents (physicians E and F). In atherothrombotic and lacunar infarction, the physician and AI software measurements had a low level of agreement.

**Figure 4:**
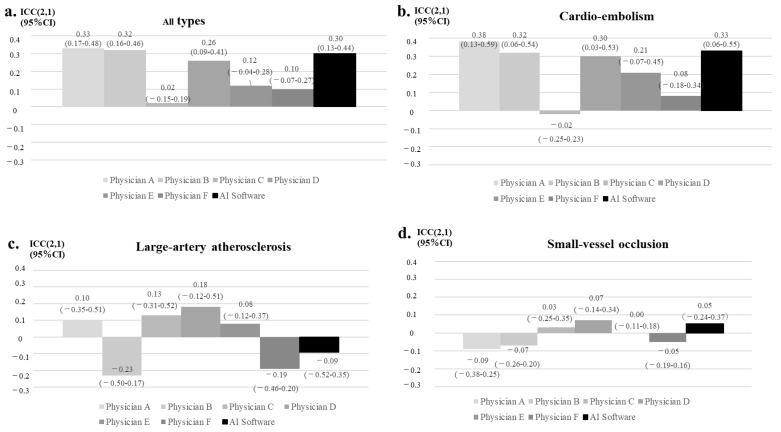
Reading accuracies of the AI software and neurologists in patients who had undergone CT images within 4.5 h from onset. The reading accuracies of the AI software and neurologists against the BS criteria for all types of ischemic stroke patients (**a**), for cardio-embolism (**b**), for large-artery atherosclerosis (**c**), and for small-vessel occlusion (**d**). The type of physician is shown on X axis; board-certified vascular neurologists (physicians A and B), neurology fellows (physicians C and D), and neurology residents (physicians E and F). Inter-assessor reliability of the total ASPECTS is shown in *Y* axis.

In population #2, the AI software had a better trend compared with the neurology residents (physicians E and F) but was at a level comparable to the board-certified vascular neurologists (physicians A and B) and one of the neurology fellows (physicians D) (Figure 4a). According to the type of ischemic stroke, the highest rate of agreement was observed for cardiogenic cerebral embolism (Figure 4b–d). In cardiogenic cerebral embolism, the results of the AI software were comparable to those of the board-certified vascular neurologist (physicians A and B) and one of neurology fellows (physicians D). In this setting, the AI results were better than those of neurology residents (physicians E and F). In atherothrombotic and lacunar infarction, the physician and AI software measurements had a low level of agreement.

### 3.3. Secondary Outcome

Next, we examined the usefulness of the AI software in determining the indication for intravenous rt-PA therapy in population #2 who had undergone CT scans within 4.5 h from onset (Table 2). In identifying patients without extensive infarction (ASPECTS of ≥6), the AI software showed an equivalent sensitivity and higher specificity compared to those of the board-certified vascular neurologist (physicians A and B).

## 4. Discussion

### 4.1. Characteristics of the AI Software

This study investigated the usefulness of automated ASPECT scoring using AI in 448 patients with acute ischemic stroke and in 132 patients who had undergone head CT imaging within 4.5 h of onset. The results showed that the accuracy of the AI software was comparable to that of board-certified vascular neurologists, and that the detection rate of cardiogenic cerebral embolism was better than that of atherothrombotic cerebral infarction. Furthermore, the specificity of the AI software in determining an ASPECTS of ≥6 was higher than that of expert physicians. Therefore, AI software may be helpful in determining the indication for intravenous rt-PA therapy.

The automated APECTS scoring for acute ischemic stroke has been reported using several other AI softwares, such as Syngo.via Frontier ASPECT Score Prototype V2 (Siemens Healthineers, Erlangen, Germany), Brainomix e-ASPECTS^®^ (Brainomix Ltd., Oxford, UK) and RAPID ASPECTS (iSchemaView, Inc., Menlo Park, CA, USA). Although the reading accuracy of these AI systems was shown to be comparable to that of physicians [10,11,12,13,14,15,16,19], these reports were limited to a population of relatively severe and extensive infarction, with the majority of cases including clot retrieval therapy and LVO [14,15,16]. In addition, neither physicians nor the AI software had a high reading accuracy for infarcts in the perforating branch region, where relatively mild cases are common [19]. Importantly, the current study analyzed the accuracy of AI software (Abierto Reading Support Solution for Stroke) on patients who had suffered all types of ischemic stroke. We showed that the detection rate of the AI software was higher for cardio-embolism infarction than for atherothrombotic and lacunar infarctions (Figure 3). Thus, these characteristics would be common among AI software for stroke diagnoses.

In Japan, relatively mild cases of cerebral infarction are often observed, with 50% of patients presenting with an NIHSS score of less than 4 and 75% with an NIHSS score of less than 9 [4]. Therefore, this study was conducted in a patient population with a median NIHSS score of 3 and with many mild cases. The reading accuracy of the AI software was not high compared to those in previous studies, partly due to the inclusion of patients with mild disease types. However, the accuracy of the AI software was comparable to that of board-certified vascular neurologists who routinely read head CT images of patients with cerebral infarction. Recently, the usefulness of AI software in reading the CT images for posterior circulation area has also been demonstrated [20]. Therefore, together with AI software for posterior circulation area, the AI-based automated ASPECT scoring software could be used as a supportive tool in evaluating patients with cerebral infarction.

### 4.2. Clinical Applications of This AI Software

Regarding the application of the AI software in actual clinical practice, it is important to analyze whether it can be utilized to appropriately identify patients indicated for intravenous rt-PA therapy. Since the reading specificity of AI software in cases with an ASPECTS of ≥6 was more likely to be higher than that of board-certified vascular neurologists (Table 2), the advantage of this AI software is that it could exclude patients with contraindications to intravenous rt-PA therapy more systematically than experts. One disadvantage is that the sensitivity of the AI software was more likely to be lower than that of neurologists, suggesting that a certain number of cases with an ASPECTS of ≤5 based on the AI software may include patients who were originally thought to have indications for intravenous rt-PA therapy. These results indicate that AI software can select the patients more safely and could be a supportive tool in clinical practice. Therefore, even in hospitals without a board-certified vascular neurologist on staff, where intravenous rt-PA therapy has not been available up to now, AI software may increase the possibility of selecting patients with an indication for intravenous rt-PA therapy. As a result, the AI software will be valuable if it can increase the number of cases in which drip and ship can be performed, especially in medically underpopulated areas.

**Figure 5:**
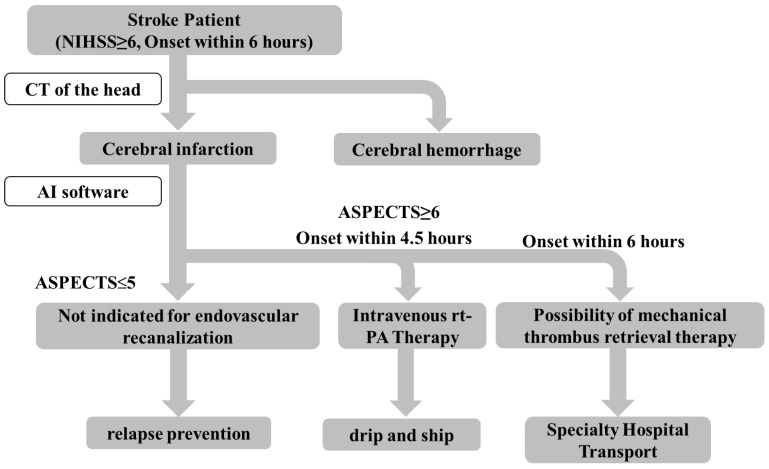
Possible diagnostic algorithm of stroke utilizing the AI software.

Based on the abovementioned data, we developed a diagnostic algorithm for stroke (NIHSS ≥ 6, onset within 6 h) utilizing AI software (Figure 5). After hospital arrival, patients with cerebral hemorrhage and subarachnoid hemorrhage were first excluded by utilizing the cerebral hemorrhage determination AI software [17] or contraindications to rt-PA treatment based on blood tests and other tests. After excluding patients with hemorrhagic lesions, if the AI software determined that the ASPECTS was ≥6, intravenous rt-PA therapy and the drip and ship method would be considered, especially for patients with high probability of cardioembolic stroke. By contrast, if the software indicated an ASPECTS of ≤5, or if the patient was not amenable to a timely acute treatment, there would be minimal need for an urgent hospital transfer. Hence, general cerebral infarction treatment would be recommended. This algorithm may be supportive for non-expert physicians at facilities without board-certified vascular neurologists by reducing the burden of image reading, thereby hopefully increasing the number of cases in which intravenous rt-PA therapy is appropriately administered. As a result, the number of patients who are transported to a tertiary care hospital may increase.

Our study had several limitations. As we included only Japanese people, the generalizability of our findings may be limited. Furthermore, we did not perform a second read to compare ASPECTS before and after using the software by the same person. Hence, we were not able to examine how the AI software affected reading. Moreover, the size of population #2, which included the patients within the intravenous rt-PA administration window, was small. We need to analyze the efficacy of AI software in this population using a larger number of patients in a future study. In addition, all the physicians who read the images for the verification were neurologists and not general physicians. We will conduct a similar study in a medically underpopulated area in the future.

## 5. Conclusions

In patients with acute cerebral infarction, the reading accuracy of the AI software was comparable to that of board-certified vascular neurologists. Furthermore, it may be helpful in determining whether intravenous rt-PA therapy is indicated. Further pivotal studies to determine the usefulness of this AI software with a large numbers of stroke patients in a medically underpopulated area would be warranted.

## Figures and Tables

**Table 1:** Characteristics of the patients.

Characteristics	Population #1 (*n* = 448)	Population #2 (*n* = 132)
Age, Mean (SD)	73.8 (13.2)	74.8 (13.0)
Sex (Male), *n* (%)	272 (60.7)	76 (57.6)
Paralysis		
Right palsy, *n* (%)	202 (45.1)	64 (48.5)
Left palsy, *n* (%)	162 (36.2)	43 (32.6)
Unknown, *n* (%)	84 (18.8)	25 (18.9)
Headache, *n* (%)	7 (1.6)	1 (0.8)
NIHSS		
NIHSS score median (IQR)	3 (1–8)	4.5 (1–12)
NIHSS ≤ 5, *n* (%)	300 (67.0)	75 (56.8)
6 < NIHSS ≤ 15, *n* (%)	95 (21.2)	30 (22.7)
16 ≤ NIHSS, *n* (%)	53 (11.8)	27 (20.5)
Disease Type		
Small-vessel occlusion, *n* (%)	137 (30.6)	29 (22.0)
Large-artery atherosclerosis, *n* (%)	96 (21.4)	18 (13.6)
Cardio-embolism, *n* (%)	102 (22.8)	53 (40.2)
Others, *n* (%)	113 (25.2)	32 (24.2)
rt-PA, *n* (%)	20 (4.5)	20 (15.2)
Endovascular therapy, *n* (%)	4 (0.9)	4 (3.0)
Time from onset to CT (hours)	18.9 ± 18.3	2.4 ± 1.0
Infarct area		
Forward Circulation, *n* (%)	331 (73.9)	99 (75.0)
Forward + Backward Circulation, *n* (%)	18 (4.0)	6 (4.5)
Backward Circulation, *n* (%)	99 (22.1)	27 (20.5)

**Table 2:** Sensitivity and specificity of physicians and the AI software.

Accuracy	Physician A	Physician B	Physician C	Physician D	Physician E	Physician F	AI Software
Sensitivity	0.99	0.98	0.98	0.98	0.98	1.00	0.93
Specificity	0.20	0.20	0.00	0.10	0.10	0.10	0.30

## Data Availability

The data presented in this study are available on request from the corresponding author. The data are not publicly available due to privacy protection.
